# A protocol for rapid construction of senescent cells

**DOI:** 10.3389/fnint.2022.929788

**Published:** 2022-07-29

**Authors:** Xing Yu, Jing Quan, Shuai Chen, Xinyue Yang, Shuai Huang, Gang Yang, Yujing Zhang

**Affiliations:** ^1^Key Laboratory of Molecular Epidemiology of Hunan Province, School of Medicine, Hunan Normal University, Changsha, China; ^2^Key Laboratory of Model Animals and Stem Cell Biology of Hunan Province, School of Medicine, Hunan Normal University, Changsha, China; ^3^Department of Breast and Thyroid Surgery, Yiyang Central Hospital, Yiyang, China

**Keywords:** aging, cellular senescence, senescent cell models, biomarker, SASP

## Abstract

Aging may be the largest factor for a variety of chronic diseases that influence survival, independence, and wellbeing. Evidence suggests that aging could be thought of as the modifiable risk factor to delay or alleviate age-related conditions as a group by regulating essential aging mechanisms. One such mechanism is cellular senescence, which is a special form of most cells that are present as permanent cell cycle arrest, apoptosis resistance, expression of anti-proliferative molecules, acquisition of pro-inflammatory, senescence-associated secretory phenotype (SASP), and others. Most cells cultured *in vitro* or *in vivo* may undergo cellular senescence after accruing a set number of cell divisions or provoked by excessive endogenous and exogenous stress or damage. Senescent cells occur throughout life and play a vital role in various physiological and pathological processes such as embryogenesis, wound healing, host immunity, and tumor suppression. In contrast to the beneficial senescent processes, the accumulation of senescent also has deleterious effects. These non-proliferating cells lead to the decrease of regenerative potential or functions of tissues, inflammation, and other aging-associated diseases because of the change of tissue microenvironment with the acquisition of SASP. While it is understood that age-related diseases occur at the cellular level from the cellular senescence, the mechanisms of cellular senescence in age-related disease progression remain largely unknown. Simplified and rapid models such as *in vitro* models of the cellular senescence are critically needed to deconvolute mechanisms of age-related diseases. Here, we obtained replicative senescent L02 hepatocytes by culturing the cells for 20 weeks. Then, the conditioned medium containing supernatant from replicative senescent L02 hepatocytes was used to induce cellular senescence, which could rapidly induce hepatocytes into senescence. In addition, different methods were used to validate senescence, including senescence-associated β-galactosidase (SA-β-gal), the rate of DNA synthesis using 5-ethynyl-2′-deoxyuridine (EdU) incorporation assay, and senescence-related proteins. At last, we provide example results and discuss further applications of the protocol.

## Introduction

Aging, a near-universal feature of organisms, could be defined as the irreversible proliferative deterioration related to the time of physiological processes of organisms that is beneficial for their survival and fertility (Hernandez-Segura et al., 2018). For most species, aging induces a variety of degenerative pathologies characterized by multiple chronic diseases, frailty, and loss of independence (Zinger et al., 2017). The aging of the world’s population is growing rapidly, which further threatens social and economical stability. One of the huge challenges of biomedical research is to shorten, if not eliminate, the period of aging and increase the health life span. Until recently, the profound association between aging and chronic disease has been noticed with a little hope of intervention. There is no doubt that it is crucial in clinical practice to break the link between the basic aging processes and chronic diseases, making aging as a modifiable factor based on the elucidation of aging-related mechanism. There is mounting evidence that aging-related dysfunctions may be driven by one or more fundamental processes which has inspired efforts to investigate these processes and find new strategies or pharmacological interventions (Calcinotto et al., 2019).

One of the main basic processes closely associated with aging-related disorders is cellular senescence (Calcinotto et al., 2019). Cellular senescence refers to irreversible growth arrest that occurs in culture or *in vivo* as reaction to the inordinate intracellular and extracellular stress (Kowald et al., 2020). It was first described by Hayflick and Moorhead (1961) approximately five decades ago when showed that the human fibroblast cells (WI-38 fibroblasts) reached their permanent arrest after 50 populations of cell division, losing ability to proliferate in culture. Despite encountering some skepticism at the time, the research of cellular senescence has begun. To date, three types of cellular senescence have been identified based on the causes of its occurrence. Replicative senescence (RS) is the first cellular senescence to be discovered by Hayflick in 1961 related to proliferation limit, due to telomere shortening (the so-called “ledomere shoDNA end replication problem”) (Hayflick and Moorhead, 1961). In addition, cells may become senescent in response to a variety of stimulation, being named as stress-induce premature senescence (SIPS) (Childs et al., 2015; Zhang et al., 2021). Currently, different stressors have been detected, including but not limited DNA damage (independent of telomere shortening), oncogenic, oxidative, and genotoxic sources. RS and SIPS seem to relay on the same pathways to induce cell cycle arrest, such as p53-p21^*CIP*1^ and p16^*Ink*4a^- retinoblastoma protein (pRB) for the inhibiting of E2F (Loo et al., 2020). However, there is a great difference between the both, that is, shorter time required for cell cycle arrest in SIPS and the shorter telomeres in RS (von Kobbe, 2018). Interestingly, developmentally programmed senescence (DPS) is the latest cellular senescence pathway to be described, which has been first identified in the first days of embryogenesis, and is characterized by p21-dependent cell cycle arrest. While neither DNA damage nor the activation of DNA damage-dependent ATM or ATR was detected in DPS.

Permanent cell cycle arrest is the essential characteristic of senescent cells. In addition, the changes in morphology and some molecular markers are also used to identify senescent cells. Even so, no single feature is specific to cellular senescence, including cell growth arrest (Ogrodnik, 2021). Similarly, not all the senescent cells display the markers of cellular senescence identified so far. Therefore, the senescent cells are often determined by a collection of markers, such as cell enlarged and flattened morphology, senescence-associated heterochromatin foci (SAHF), the absence of proliferating cell nuclear antigen (PCNA) protein, and activity of senescence-associated-galactosidase (SA-β-gal). In addition, the senescent cells are characterized by G1 or G2/M phase arrest that are no longer to be divided. Another important feature of the senescent cells is senescence-associated secretory phenotype (SASP), which may further induce senescence-related inflammation, aging phenotypes, chronic diseases, or loss of resilience (Pang et al., 2017; Loo et al., 2020; Sikora et al., 2021).

The senescent cells could secrete pro-inflammatory cytokines, growth factors or proteases, which are all collectively known as SASP (Birch and Gil, 2020). Pro-inflammatory factors such as interleukin-1 (IL-1), interleukin-6 (IL-6), and interleukin-8 (IL-8) are the key components of SASP. Moreover, other proteins are closely associated with the SASP of cells undergoing senescence, including IGF-binding proteins (IGFBPs), CCL-2 (MCP-1), CCL-5 (RANTES), CCL-7 (MCP-3), CXCL-1, and CXCL12 (SDF-1), significantly increase in a variety of aging tissues and occur together with sterile inflammation. For example, IL-6 and IGFBP2 are much lower in the non-senescent cells isolated from the fat tissue of young mice than the senescent cells from the same tissue. Similarly, our previous research also identified the increased level of IL-6 in the premature senescent cells induced by hexavalent chromium (Zhang et al., 2022). Interestingly, the primordial function of SASP is to recruit the immune cells to remove senescent cells, but it could propagate from cell to cell potentially aggravating the senescent cell burden of chronic disease progression when the ability of immune cells to clear senescent cells is overcome; thus, it can be seen that the secretion of may further influence microenvironments and cause senescent phenotypes in the surrounding normal cells.

## Protocol

### Materials and reagents preparation

1. Cell culture plates (NEST).
2. L02 hepatocytes (a gift from Nanjing Medical University).
3. RPMI 1640 (Gibco) with 10% Fetal bovine serum (FBS, Gibco) and 1% Penicillin-streptomycin (1,000 U/ml, Gibco).
4. About 0.25% Trypsin-EDTA (Gibco).
5. RIPA lysis buffer (Beyotime Technology).
6. Phosphate-buffered saline (PBS, Beyotime Technology).
7. Antibody against TriMethyl-Histone H3-K9 (ABclonal Technology).
8. Antibody against PCNA (Cell Signaling Technology).

## Methods

### Replicative senescence

1. Thaw low-passage L02 hepatocytes

NOTE: In the present protocol, the L02 hepatocytes were used, but this protocol could be used in other cell types, such as endothelial or epithelial cells. In different cell types, cell culture conditions need to be optimized according to the growth rate and characteristics.

2. Calculate the population doubling level (PDL) at each passage according to the formula: PDL = log (*N*f/*N*0)/log2. Nf refers to the final cell number and N0 refers to the initiate cell number that is the number of seeded cells.

NOTE: Make sure the PDL is less than 30 at the start of experiments to avoid the presence of the senescent cells.

3. Culture L02 hepatocytes in RPMI 1640 medium with 10% FBS and 1% penicillin/streptomycin.
4. All the cells were incubated in the cell incubator at 37°C with 5% CO_2_.
5. Under the conditions, the cells were split every 2∼4 days when the cells reach 70∼80% confluence for 20 weeks until the cells were characterized by no change in PDL from one subculture to the next.

NOTE: The time at which replicative senescence occurs depends on cell types. In general, cells present replicative senescence when reaching 55∼60 generations.

### Induced replicative senescence

1. Replicative senescent cells were seeded in 100 mm tissue culture plates at a concentration of 1 × 10^6^ and incubated overnight in the cell incubator overnight.
2. The medium was removed and replaced for 10 ml fresh medium. Then, the cells were incubated for another 48 h in normal conditions.
3. The culture supernatant was collected in a 15 ml tube.
4. Centrifuge the supernatant at 1,000 g, 10 min.
5. Mix the supernatant with fresh medium at the ratio of 1:2, which was then used to culture cells with low PDL.
6. All the cells were incubated in the cell incubator at 37°C with 5% CO_2_.
7. Under the conditions, the cells were cultured continuously for about 7 weeks and split every 2∼4 day when the cells reach 70∼80% confluence. At that time, the cells show no obvious proliferation.

### The secretion of typical senescence-associated secretory phenotype (interleukin-1, interleukin-6, and interleukin-8)

1. Cells were seeded in 60 mm tissue culture plates at a concentration of 3 × 10^5^ and incubated overnight in the cell incubator overnight.
2. The medium was removed and replaced for 4 ml fresh medium. Then, the cells were incubated for another 48 h in normal conditions.
3. The culture supernatant was collected in a 15 ml tube on ice.
4. Centrifuge the samples at 300 g for 5 min and keep in –80°C until processed.
5. The ELISA was performed following the manufacturer’s instructions.

NOTE: Other methods, such as Western Blot, could be used to detect SASP protein expression. The protein levels of SASP factors are closely related to cell type.

### Senescence-associated β-galactosidase activity assay

1. The cells that have been treated were seeded in 6-well tissue culture plates at a concentration of 2 × 10^5^ cells/well to ensure that the cells are sparse. Cells were incubated overnight at 37°C, 5% CO_2_.
2. Phosphate-buffered saline were used to wash cells for 1 min with three times.
3. Cells were fixed by 4% paraformaldehyde for 15 min at room temperature and washed with PBS for three times.
4. Cells were stained by fresh staining solution (1 ml/well) prepared according to the number of samples to stain at 37°C in a dry incubator (without CO_2_ as it may affect the PH) for 12∼14 h. Significantly, some cell types may need a shorter incubation time.

NOTE: The plates need to be sealed by parafilm to avoid evaporation that may induce the formation of crystals and interfere with the observation under the microscope.

5. The stained cells were washed with PBS for two times.
6. Observe and evaluate the results. Cells are positive if the cells show a blue perinuclear under a normal light microscope.
7. Quantitative analysis. Observe 200 cells/sample and count the positive cells. The percentage of premature senescent cells was measured using the following equation: percentage of premature senescent cells = (positively stained cells/total cells) × 100%. The cells were counted manually instead of using the automated counting instrument. Compare the results of senescent cells with appropriate control.

### Senescence-associated heterochromatin foci assay

NOTE: Most senescent cells often have condensed region of DNA/chromatin that may further inhibit the expression of proliferation-promoting genes. SAHF could be detected by DAPI staining.

1. Put a coverslip/well in a 12-well tissue plate and seed 6 × 10^4^ cells one well of the plate. Then, the cells were incubated in the cell incubator at 37°C with 5% CO_2_ overnight.
2. Phosphate-buffered saline was used to wash the cells for two times.
3. The cells were fixed by 4% paraformaldehyde for 20 min at room temperature.
4. Phosphate-buffered saline was used to wash the cells for two times.
5. DAPI (1 mg/ml) was used to stain the cells for 15 min at 37? in dark.
6. After washing with PBS for two times, lift the coverslip with the aid of a pair of tweezers and a needle and let it onto glass slides. Cells were observed by the fluorescent microscopy with a peak excitation wave length at 340 nm.
7. The fluorescent intensity was quantified by Image J software.

## The mRNA and protein expression of proliferating cell nuclear antigen

### qRT–PCR for mRNA levels

1. The primer was designed to detect LMNB1: 5′-CAAAGCGGAAGAGGGTTG-3′; 5′-TTACCCTCCGACCCTCTA-3′. PCNA: 5′-TCAAGAAGGTGTTGGAGGCA-3′; 5′-TCGCAG C GGTAGGTGTCG-3′. ACTB: 5′-CACCAGGGCGTGATGGT-3′; 5′-CTCAAACATGATC TGGGTCAT-3′.
2. The cells were seeded in 60 mm culture tissue plate at a concentration of 5 × 10^5^ and incubated in the cell incubator at 37°C, 5% CO_2_ overnight.
3. The total RNA was isolated by TRIzol reagent and the cDNAs were synthesized by sensiscript RT Kit.
4. The qRT–PCR reaction mi × was prepared according to Table 1.
5. All the simples need to be run in duplicate.
6. Cover the plate with a seal and run the plate in Real-Time PCR Detection System (ABI7300 plus) following the protocol: 95°C for 30 s, 40 cycles of 95°C for 5 s, and 60°C for 34 s.
7. Use *ACTB* as reference genes to calculate the Δ Ct value and use the appropriate control to calculate the ΔΔ Ct value.

**TABLE 1:** Composition of the qRT–PCR reaction mix.

Component	Volume (μl)
SYBR Green	4.0
Forward primer (10 mM)	0.4
Reverse primer (10 mM)	0.4
Nuclease-free water	4.2
cDNA (∼5 ng)	1.0
Total	10.0

### Western blot for protein levels

1. About 2 × 10^5^ cells were seeded in 6-well tissue culture plates and incubated in the cell incubator overnight.
2. Phosphate-buffered saline was use to wash the cell for two times.
3. The cells were lysed with 120∼150 μl RIPA lysis buffer with 1 mM phosphatase inhibitors (cocktail and PMSF) for 30 min on ice.
4. Centrifugate the samples at 15,000 g for 20 min at 4°C and remove the supernatant into a new tube on ice.
5. The Bradford protein assay was used to quantitate protein.
6. Take out 20∼25 μl of lysate and add to 5∼6.25 μl 5 × SDS and mi × well.

NOTE: The volume ratio of lysate to 5 × SDS is 1:4.

7. The samples were boiled at 100°C on a heat block for 5 min.
8. After running in 5∼15% SDS–PAGE gels (60 V for 30 min, 120 V for 45∼55 min), the gel was transferred to NA membrane (100 V for 90 min).
9. The membrane was blocked for 60 min at room temperature with 5% non-fat milk in TBS with 0.1% Tween 20 and incubated overnight at 4°C with the primary antibody of PCNA and GAPDH.
10. The primary antibodies were incubated with horseradish peroxidase conjugated secondary antibody for 1 h at room temperature, and immunoreactive bands were detected using an ECL kit.
11. Image J software was used to analyze the density of the immunoreactive bands.

### Ethynyl-2′-deoxyuridine staining

1. Put a coverslip/well in a 12-well tissue plate and seed 6 × 10^4^ cells one well of the plate. Then, the cells were incubated in the cell incubator at 37°C with 5% CO_2_ overnight.
2. The cells were incubated with EdU working fluid (10 μM) at 37°C for 4 h.
3. Phosphate-buffered saline was used to wash the cells for two times.
4. The cells were fixed by 4% paraformaldehyde for 20 min at room temperature and incubated with 5 mg/ml glycine for 5 min.
5. The cells were permeablized with 0.5% Triton -100 in PBS for 20 min at room temperature following washed twice with 3% bovine serum albumin (BSA) in PBS.
6. About 3% BSA in PBS was used to wash the cells for three times.
7. The cells were stained with a Click-iT reaction cocktail containing for 30 min away from light.
8. About 3% BSA in PBS was used to wash the cells for three times.
9. Hoechst 33342 (5 μg/ml) was used to stain DNA for 30 min at room temperature.
10. Phosphate-buffered saline was used to wash the cells for two times.
11. Lift the coverslip with the aid of a pair of tweezers and a needle and let it onto glass slides. Cells were observed by fluorescent microscopy.

## Representative result

### Typical senescence-associated secretory phenotype increased in the supernatant from replicative senescent cells

**FIGURE 1:**
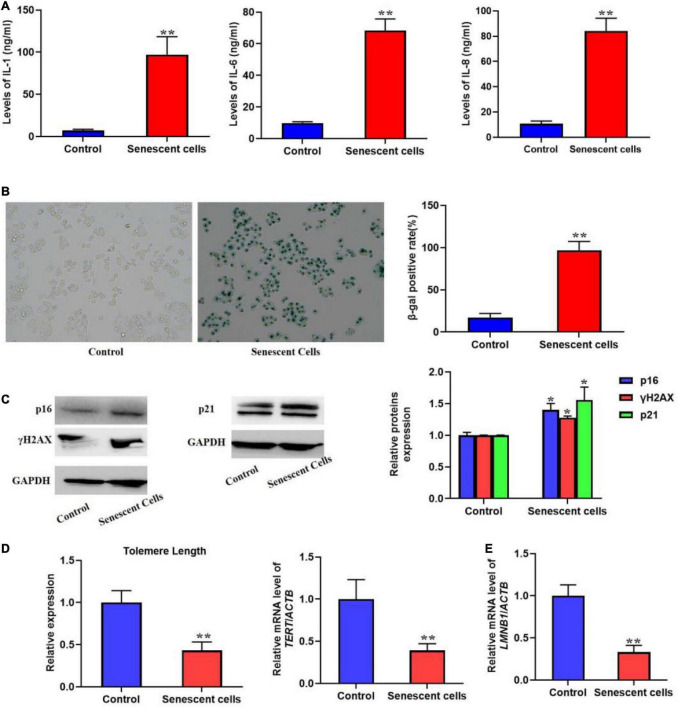
Replicative senescent cells secreted SASP. The L02 hepatocytes were cultured for 20 weeks. **(A)** The secretion of IL-1, 6, and 8 were significantly increased in the supernatant. **(B)** The SA-β-gal activity was elevated. **(C)** The protein expression of p16, p21, and γH2AX. **(D)** The change of telomere length. **(E)** The mRNA level of *LMNB1*. All the data in this article were from three independent experiments (*N* = 3). *Compared with control, *p* < 0.05; **compared with control, *p* < 0.01. The control cells of replicative senescent cells were young cells with lower PDL (< 30) ensuring there is no replicative senescence.

Most senescent cell could secret various factors, especially pro-inflammatory factors, e.g., interleukins and chemokines. Previous studies also indicated that SASP could promote cellular senescence the adjacent cells of senescent cells. In the present protocol, we detected several typical pro-inflammatory factors such as IL-1, 6, and 8 using ELISA kits (Figure 1A). The result showed that in the replicative senescent cells of L02 the three significantly increased. The activity of SA-β-gal was also increased significantly, accompanied with the elevated protein levels of p16, p21, and γH2AX (Figures 1B,C). Additionally, the results in Figures 1D,E showed the shorten telomere length and down-regulated LMNB1 mRNA levels. These results indicted the senescent L02 hepatocytes secreted SASP.

### The supernatant from replicative senescent cells induced cellular senescence

**FIGURE 2:**
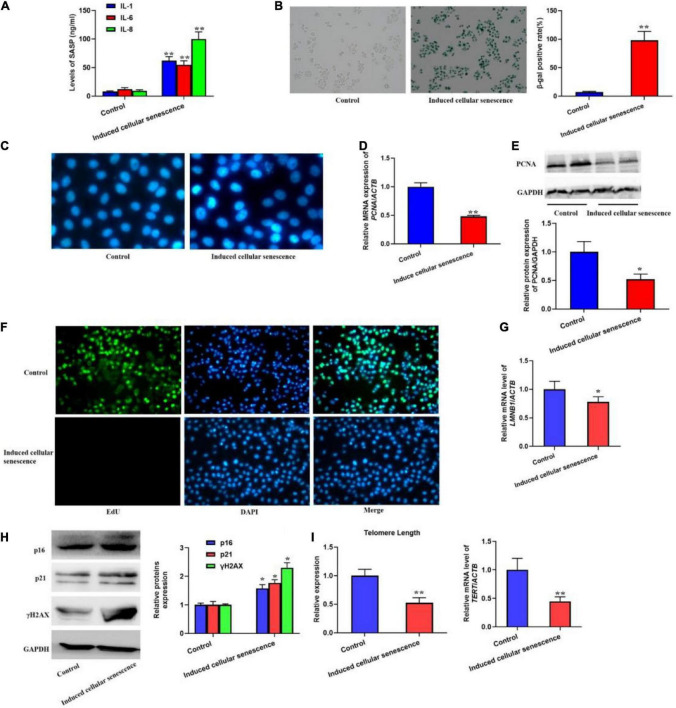
The supernatant from replicative senescent cells induced cellular senescence. The cells were cultured by the supernatant from senescent cells for 6 weeks. **(A)** The secretion of IL-1, 6, and 8 were significantly increased in the supernatant. **(B)** The SA-β-gal activity was elevated. **(C)** The SAHF formation was increased. The decreased cell proliferation with downregulated expression of PCNA and EdU staining. **(D)** The relative mRNA expression of PCNA. **(E)** The protein levels of PCNA. **(F)** EdU staining. **(G)** The mRNA level of *LMNB1*. **(H)** The protein expression of p16, p21, and γH2AX. **(I)** The change of telomere length. All the data in this article were from three independent experiments (*N* = 3). *Compared with control, *p* < 0.05; **compared with control, *p* < 0.01. The control of induced senescent cells is the L02 hepatocytes cultured by RMPI 1640 medium with 10% FBS and 1% penicillin-streptomycin for 7 weeks.

In order to investigate if SASP secreted by the senescent cells could induce cellular senescence, we used the supernatant from the senescent cells to culture L02 hepatocytes for 7 weeks. The results should be that the secretion of IL-1, 6, and 8 was significantly increased (Figure 2A). Meanwhile, we also found the elevated SA-β-gal activity and SAHF formation (Figures 2B,C). In addition, we also detected the biomarker of cell proliferation, including PCNA protein level and the EdU staining (Figures 2D–F), which presented decreased expression of PCNA and EdU incorporate. In addition, the mRNA level of LMNB1 was was decreased (Figure 2G). Furthermore, the expression of p16, p21, and γH2AX were also remarkably increased, accompanied with shorten telomere length (Figures 2H,I). Altogether, results confirmed the SASP from senescent cells could rapidly induce cellular senescence.

### Statistical analysis

All the results were presented as the mean ± SD from three independent experiments unless otherwise indicated. Significant difference among the groups was evaluated by analysis of one-way ANOVA. *Post-hoc* test was analyzed by the Student–Newman–Keuls (SNK) test. Differences were considered significant at *P* < 0.05. All the statistical analyses were performed using the Statistical Program for Social Sciences (SPSS), version 20.0.

## Discussion

We describe here a new method for rapid induction of replicative cellular senescence in hepatocytes. Culture supernatant derived from replicative senescent cells is essential for the rapid method. The protocols could be widely used to construct replicative cellular senescence model in other cell types account to it dramatically shortens the time it takes for cellular senescence to occur. In addition, our protocol also provided the representative results of different senescence markers to confirm cellular senescence because some indicators could be regulated by various normal physiological or pathological conditions that may or may not relate to the senescent cells.

It has been reported that senescent cells often secrete various proteins with potent effects, including cytokines, chemokines, growth factors, or MMPs (Wiley and Campisi, 2021). As our previous research found that the levels of IL-6 and FGF23 were profoundly increased in the hexavalent chromium-induced premature senescent hepatocytes (Zhang et al., 2022). Similarly, parkinsonian SNpc tissues displayed elevated protease MMP-3 and pro-inflammatory cytokines IL-6, IL-1α, and IL-8 (Chinta et al., 2018). Increased MMP3 expression also presented upregulation in cortical tissue in patients with AD (Bhat et al., 2012). Interestingly, SASP has dual roles in the cellular senescence. At first, the SASP is regarded as an inhibitor of cellular senescence *via* recruiting immune cells to clear senescent cells termed “senescence surveillance” (Loo et al., 2020). In addition to the function, prolonged secretion of SASP is closely involved in sensitizing non-senescent adjacent cells to senescence. In contrast to the beneficial effects of SASP, this function is mainly related to the accumulation of senescent cells, depending on the declined immune function that leads to failures in senescence surveillance (Birch and Gil, 2020). In our previous study, we selected SASP as the marker of premature senescence of L02 hepatocytes caused by Cr(VI) (Zhang et al., 2016). Furthermore, the study performed by our lab has indicated that the ROS-calcium-NF–κB-signaling pathway could regulate the levels of FGF-23 and IL-6 involved in the Cr(VI)-induce premature senescence (Zhang et al., 2022). In the present protocol, we found the significantly elevated expression of IL-1, IL-6, and IL-8 in the supernatant of the replicative senescent cells. Based on the role of SASP in inducing cellular senescence on the senescent cells neighboring cells by changing microenvironment, we in the protocols used the conditioned medium from senescent cells to rapidly induce cellular senescence. In addition, multiple senescence markers were selected to assess the senescent cells together in the present protocols, including SA-β-gal, SAHF foci, and the cell proliferation marker PCNA and EdU staining. There is a growing need for further research to investigate how the different signaling pathways regulating SASP in cellular senescence due to the pleiotropic effects of these factors in senescence. At present, studies suggested DDR, NF–κB/CEBPβ, mTOR signaling, autophagy, etc., were the possible mechanism of SASP in the cellular senescence (Kang and Elledge, 2016; Birch and Gil, 2020). However, these mechanisms related to the induced senescent cells in the present study need to be further investigated.

The activity of SA-β-gal is commonly used to evaluate the elevated β-gal activity in the senescent cells related to enhanced lysosomal activity (Jannone et al., 2020). SA-β-gal, present in lysosomes of senescent cells, is the isoform of β-gal enzyme that is responsible for the breakdown of β-gal. Therefore, the increased activity of is SA-β-gal is normally considered as the outcome of senescence. Staining for SA-β-gal, as described here, we fixed the cell by 4% PFA and obtained the increased activity of SA-β-gal in the senescent cells. Although the methods have been developed to assess SA-β-gal activity using a fluorogenic substrate for β-gal activity (Cazin et al., 2017), we did not use the technique to detect the marker. In the future research, we will try the new protocols to determine the β-gal activity for identifying cellular senescence.

Although the elevated activity of β-gal is regarded as the widely used biomarker for senescent cells, it is not the exclusive marker of senescence (You et al., 2019). Therefore, it is essential to detect other independent canonical markers to confirm cellular senescence in our study. We examined the formation of SAHF in nucleus by staining with DAPI because chromatin reorganization also occurs in the senescent cells. Herein, we found the formation of SAHF in induced replicative senescence. However, it should be noted that SAHF may not be presented in all the senescent cells ascribed to its formation is closely related to cell line. In addition to SAHF observation, the PCNA and EdU staining closely associated with cell proliferation are also to be detected in the present study. These results showed decreased expression of PCNA and fewer cells incorporate EdU after cellular senescence. Taken together, all these results confirmed that culture with supernatant from replicative cells could rapidly induce cellular senescence. While this protocol is focused on hepatocytes, it could be certainly extended to other cell lines.

There is mounting evidence that senescent cells can not only cause degenerative pathology but also induce hyperplastic pathology, especially cancer. Understanding the mechanisms of how senescence involves in diseases will certainly have a tremendous effect on medicine, but it takes long time to construct cellular senescence models *in vivo* and *in vitro*. This protocol provides a significant and helpful tool to facilitate the construction and identification of cellular senescence.

## Data availability statement

The original contributions presented in this study are included in the article/supplementary material. Further inquiries can be directed to the corresponding author/s.

## Author contributions

YZ and XY proposed the study. JQ, SC, XYY, SH, and GY performed the experiments. YZ wrote the first draft. All authors contributed to the interpretation of the study and revised further drafts.
